# Catalytic Pyrolysis of Biomass: A Review of Zeolite, Carbonaceous, and Metal Oxide Catalysts

**DOI:** 10.3390/nano15070493

**Published:** 2025-03-26

**Authors:** Weiqiang Sun, Yihong Yan, Yuxin Wei, Jingjing Ma, Zhenchuan Niu, Guang Hu

**Affiliations:** 1 School of Nuclear Science and Technology, Xi’an Jiaotong University, Xi’an 710049, China; sunweiqiang@xjtu.edu.cn (W.S.); yihong@stu.xjtu.edu.cn (Y.Y.); 2 School of Human Settlements and Civil Engineering, Xi’an Jiaotong University, Xi’an 710049, China; weiyuxin5924@stu.xjtu.edu.cn (Y.W.); jingjma2022@163.com (J.M.); niuzc@ieecas.cn (Z.N.); 3 State Key Laboratory of Loess Science, Institute of Earth Environment, Chinese Academy of Sciences, Xi’an 710061, China

**Keywords:** pyrolysis, biomass, zeolites, metal oxides, carbonaceous materials

## Abstract

This review provides an exploration of various catalytic pyrolysis techniques for bio-oil production, focusing on the effects of different pyrolysis methods (slow, fast, and flash pyrolysis) on bio-oil yield and composition. The review also discusses key factors influencing bio-oil production, including feedstock composition (cellulose, hemicellulose, and lignin), and the role of catalytic materials in enhancing yield and product selectivity. Three primary classes of catalysts—zeolites, carbonaceous materials, and metal oxides—are thoroughly examined, with a discussion on the differences between bulk catalysts and nanocatalysts. The paper highlights how these catalysts influence the formation of bio-oil components such as phenols, hydrocarbons, and oxygenated compounds. Furthermore, this review discusses recent advancements in catalyst design and modifications to optimize bio-oil production. This review provides the latest advancements in catalytic pyrolysis, emphasizing the correlation between catalyst properties and the resulting products. It aims to offer valuable insights into the future potential of catalytic pyrolysis for efficient biomass conversion and sustainable biofuel production.

## 1. Introduction

Biomass, derived from plant and animal materials, is a renewable and sustainable source of energy and chemicals. As an alternative to fossil fuels, biomass offers a promising pathway for producing biofuels and bio-based chemicals while reducing environmental impacts such as greenhouse gas emissions [1]. Pyrolysis, a thermal decomposition process, is one of the most widely researched methods for converting biomass into bio-oil, biochar, and biogas [2]. Bio-oil can be upgraded into renewable fuels or valuable chemicals through hydrodeoxygenation, catalytic cracking, or emulsification, enhancing its stability and energy content. It is primarily used for combustion in boilers and turbines or as a feedstock for biofuels and chemicals. Bio-gas, composed mainly of CO, H_2_, CH_4_, and CO_2_, serves as a fuel for power generation and heating. After purification, it can be converted into hydrogen or synthetic fuels via Fischer–Tropsch synthesis [3,4]. However, the direct pyrolysis of biomass suffers from low product selectivity, particularly for valuable chemicals, due to the complexity and heterogeneity of the biomass composition [4]. This often leads to a mixture of undesirable by-products, such as acids, phenols, and other oxygenates, which limits the application of bio-oil in various industries [5].

Catalytic pyrolysis has gained significant attention as an advanced method for improving the efficiency and product selectivity of biomass conversion. Unlike conventional pyrolysis, which relies on heat alone to break down biomass, catalytic pyrolysis involves the introduction of catalysts to facilitate the decomposition of biomass at lower temperatures and with higher precision in controlling product formation [6]. The primary advantage of catalytic pyrolysis lies in its ability to enhance the deoxygenation of bio-oil, reducing the oxygen content and improving its energy density. This process not only results in the production of bio-oil with a higher hydrogen-to-carbon (H/C) ratio, but also significantly decreases the formation of unwanted by-products such as acids, aldehydes, and phenols, which are common in non-catalytic pyrolysis [7]. The introduction of catalysts can alter the chemical pathways of biomass decomposition, increasing the selectivity for valuable aromatic hydrocarbons, alkylphenols, and other bio-based chemicals. These compounds have high economic value and can be used as precursors for various industrial applications, including biofuels, plastics, and pharmaceuticals [8,9]. Catalytic pyrolysis also helps to minimize coke formation, a common issue in non-catalytic pyrolysis that results in catalyst deactivation and reduced bio-oil yields [10].

**Figure 1:**
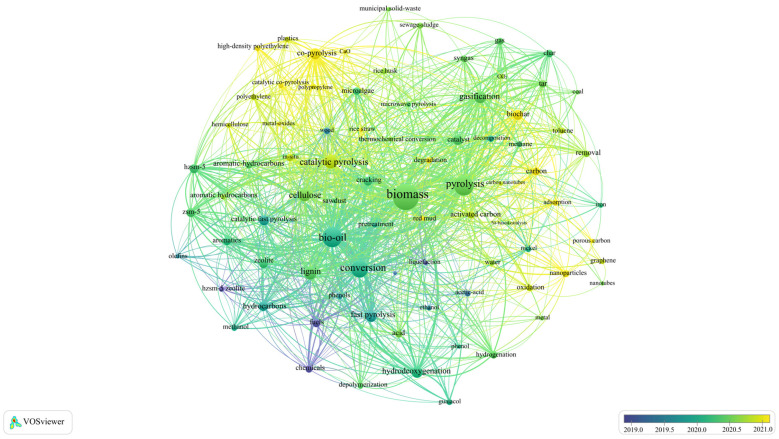
Biomass catalytic pyrolysis and its catalysts as keywords in the timeline hotspot map generated by VOS viewer.

Using “Biomass catalytic pyrolysis” and “Catalyst” as keywords on the Web of Science, 4959 articles from 2015 to 2025 were selected. Through the keyword visualization analysis by VOSviewer software (v.1.6.20), it was found that research in catalytic pyrolysis has been rapidly advancing, focusing on a variety of catalyst types (Figure 1). The most commonly studied catalysts include zeolite-based catalysts, carbonaceous materials, and metal oxide catalysts. Zeolites, with their well-defined microporous structures, are known for their shape-selectivity, enabling the selective production of aromatic compounds and olefins [11]. Carbonaceous materials, such as activated carbon (AC), offer cost-effective, recyclable options that can be modified to enhance selectivity towards specific products like phenols and hydrocarbons [12,13]. Metal oxide catalysts, including calcium oxide (CaO), magnesium oxide (MgO), and transition metal oxides, are recognized for their ability to deoxygenate bio-oil, improving its stability and calorific value [14,15].

This review focuses on the three most commonly used catalyst types in catalytic pyrolysis—zeolite-based catalysts, carbonaceous materials, and metal oxides—and examines their catalytic mechanisms, advantages, limitations, and recent advancements. By understanding the unique properties of each catalyst type and their role in improving bio-oil yield and selectivity, we aim to provide insights into their future potential for biomass conversion and the development of sustainable biofuel production technologies.

## 2. Different Types of Pyrolysis

Pyrolysis is a thermal decomposition process of organic materials in the absence of oxygen, leading to the production of biochar, bio-oil, and syngas. The specific conditions under which pyrolysis is conducted, such as temperature, heating rate, and residence time, significantly influence the distribution of these products [6]. Based on these operational parameters, pyrolysis can be categorized into several types:

### 2.1. Slow Pyrolysis

Slow pyrolysis is a thermochemical process that decomposes organic materials, such as biomass, in an oxygen-limited environment. This method is characterized by its slow heating rates, often less than 1 °C per second, and extended reaction times, allowing for gradual biomass decomposition and higher biochar yields [16]. The process involves prolonged residence times, allowing for thorough thermal degradation of the feedstock. The primary product of slow pyrolysis is biochar, a carbon-rich solid, with bio-oil and syngas as secondary products [17,18].

The distribution and characteristics of products in slow pyrolysis are shaped by several critical factors, with temperature playing a pivotal role. Typically conducted between 300 and 700 °C, this process sees a decline in biochar yield at higher temperatures as more of the biomass is converted into bio-oil and syngas. The heating rate is another crucial parameter, with slow heating—generally below 1 °C per second—favoring biochar formation by enabling a controlled breakdown of biomass components, ultimately leading to a higher solid residue. Additionally, the extended residence time, often exceeding 5 min, allows for thorough decomposition and promotes secondary reactions that enhance both the stability and yield of biochar [19]. Unlike other pyrolysis process, slow pyrolysis is also capable of processing larger biomass particles, typically ranging from 5 to 50 mm, making it suitable for a wide range of feedstocks, including woody biomass and agricultural residues, without requiring extensive pre-treatment [6].

### 2.2. Fast Pyrolysis

Fast pyrolysis is a thermochemical process that rapidly heats biomass in the absence of oxygen to produce bio-oil, biochar, and syngas. This process typically operates at temperatures between 400 and 800 °C, with heating rates of 10 to 200 °C per second, and very short vapor residence times of less than 2 s [20]. These conditions favor the formation of bio-oil, which can constitute up to 70% of the product yield. Various reactor configurations have been explored for fast pyrolysis, including vortex reactors, entrained flow reactors, rotating reactors, circulating fluidized beds, vacuum furnace reactors, wire mesh reactors, and fixed bed reactors [21,22,23,24]. Among these, fixed bed and fluidized beds have been identified as particularly effective for bio-oil production.

The rapid heating and short residence times in fast pyrolysis minimize secondary reactions, thereby maximizing bio-oil yield. The efficiency of this process is influenced by factors such as the moisture content and particle size of the biomass feedstock, as well as the pyrolysis temperature and heating rate. Optimizing these parameters is crucial for enhancing bio-oil production. The bio-oil produced through fast pyrolysis is a complex mixture of oxygenated hydrocarbons and requires further upgrading to be used as a transportation fuel. A recently developed pyrolysis technique, termed intermediate pyrolysis, bridges the gap between slow and fast pyrolysis by operating under conditions that are moderate in both heating rate and residence time. This approach offers the benefit of producing bio-oil with improved fluidity and lower tar content, making it more suitable for further processing and utilization [25].

### 2.3. Flash Pyrolysis

Flash pyrolysis is distinguished by its extremely rapid heating rates, exceeding 1000 °C per second, and ultra-short vapor residence times of less than 5 s [16]. The feedstock particle size is typically less than 0.1 mm [25]. Flash pyrolysis distinguishes itself from slow and fast pyrolysis through its exceptionally rapid heating rates and elevated operational temperatures. While slow pyrolysis employs gradual heating to produce biochar, and fast pyrolysis utilizes moderate temperatures with rapid heating to maximize bio-oil yield [26].

The choice of reactor design is crucial in flash pyrolysis to achieve the necessary rapid heating and short residence times. Fluidized bed reactors are commonly employed due to their efficient heat transfer capabilities and ability to maintain uniform temperature profiles. Additionally, finely ground biomass particles are swiftly heated as they are carried by a hot gas stream, ensuring the rapid thermal decomposition characteristic of flash pyrolysis [27].

## 3. Influential Parameters on Bio-Oil Yield

The yield and composition of bio-oil produced through catalytic pyrolysis are influenced by a range of factors, which play a pivotal role in optimizing the process for sustainable biofuel production. These factors include the characteristics of the biomass, such as its cellulose, hemicellulose, and lignin content, which affect the decomposition pathways and final bio-oil distribution. Another crucial aspect is the choice of pyrolysis mode, whether in situ or ex situ, which governs how the feedstock and catalyst interact during the process. Finally, the type of catalyst used, such as zeolite-based, carbonaceous materials, or metal oxides, has a profound impact on enhancing the yield, quality, and selectivity of bio-oil. This section will explore these key factors in detail, highlighting their effects on the overall bio-oil production and their potential for process optimization.

### 3.1. Biomass Characteristic

**Figure 2:**
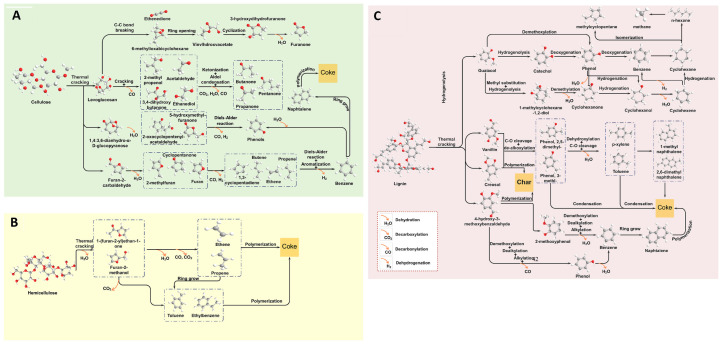
Possible decomposition pathways of lignocellulosic biomass constituents, lignin (**A**), hemicellulose (**B**), and cellulose (**C**) during the pyrolysis process [33].

Beyond operating conditions, the compositional characteristics of biomass, including cellulose, hemicellulose, and lignin, significantly influence both the yield and composition of the resulting bio-oil (Table 1). Lignocellulosic biomass consists mainly of cellulose (25–50 wt%), hemicellulose (15–40 wt%), and lignin (10–40 wt%), with other minor components. Its composition greatly influences pyrolysis and product distribution. The ratio of organic and inorganic components varies with biomass type, growth conditions, and harvesting time. Each component follows distinct pyrolysis pathways, affecting thermochemical behavior. Cellulose and hemicellulose promote bio-oil formation, while lignin favors char production. Higher lignin content increases bio-oil molecular weight and viscosity but reduces its water content [28]. As illustrated in Figure 2, the thermal decomposition of these biomacromolecules during pyrolysis leads to distinct product distributions. Cellulose, a linear macromolecule with a crystalline structure, consists of uniform polysaccharides connected by β-1,4-glycosidic bonds. Its pyrolysis process initiates with the cleavage of these bonds at temperatures ranging from 300 to 350 °C, primarily yielding furans, levoglucosan, and various oxygenated compounds such as acids, alcohols, and anhydrosugars [29]. Hemicellulose, in contrast, is made up of short-chain hetero-polysaccharides composed mainly of hexoses, pentoses, and small amounts of other saccharides [30]. The pyrolysis characteristics of hemicellulose are largely governed by the decomposition behavior of its constituent monosaccharides, acetyl groups, and uronic acids. Being less thermally stable than cellulose and lignin, hemicellulose begins to degrade at relatively lower temperatures, typically between 200 and 350 °C. The primary mechanisms governing hemicellulose pyrolysis include dehydration, depolymerization, and char formation [31].

Lignin, a complex cross-linked polymer, consists predominantly of p-hydroxyphenyl, guaiacyl, and syringyl monomers [32]. The pyrolysis of lignin mainly involves the cleavage of α-O-4 and β-O-4 ether linkages in its macromolecular structure [32]. Its thermal decomposition proceeds through alkyl chain degradation, aromatic ring transformations, and char formation, occurring over a broad temperature range of 280 to 500 °C [31]. Generally, a higher lignin content leads to increased coke formation while reducing the yield of aromatic compounds. On the other hand, biomass with greater hemicellulose content tends to generate less coke but enhances the production of non-condensable gases [7].

**Table 1:** Effects of composition characteristics of biomass on yield and composition of derived bio-oil.

Biomass	Biomass Component (%)			Biomass Yields (wt%)			Ref.
	Cellulose	Hemicellulose	Lignin	Liquid	Biochar	Biogas	
Hardwood	45–50	20–25	20–25	53.9	26.2	19.9	[34]
Hardwood	45–50	20–25	20–25	63.3	12.7	24	[35]
Softwood	35–40	20–25	27–30	45	27.6	27.4	[34]
Softwood	35–40	20–25	27–30	39.7	32.4	28.9	[36]
Lignin free	31–24	15–25	/	30	25	25	[37]

### 3.2. Effect of Pyrolysis Mode

**Figure 3:**
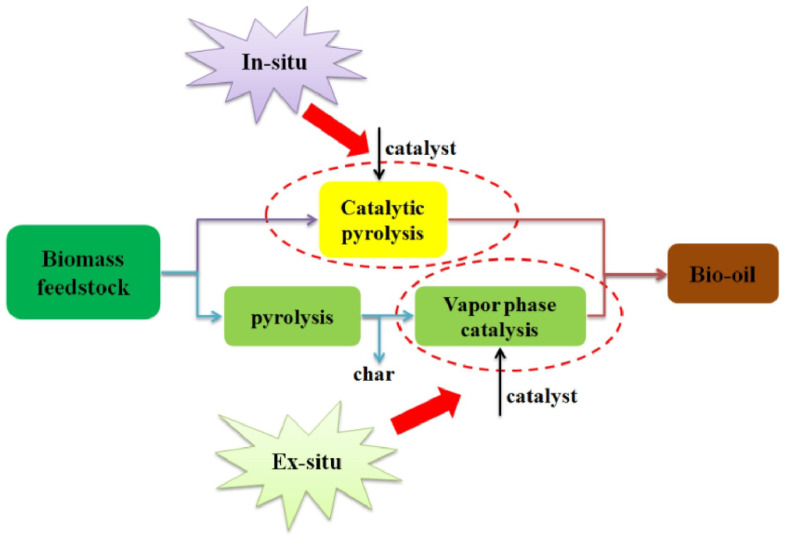
In situ and ex situ catalytic configurations [41].

Catalytic pyrolysis, an advanced method for biomass conversion, can be performed using two experimental configurations: in situ and ex situ pyrolysis. In in situ pyrolysis, the feedstock and catalyst are mixed before being introduced into the reactor, where both biomass decomposition and catalytic upgrading of the vapor occur simultaneously (Figure 3). This method allows biomass and catalyst particles to directly contact in the fluidized bed, favoring immediate cracking of the pyrolytic fragments as soon as they are released. However, challenges arise in temperature optimization, as the pyrolysis temperature is constrained, and some vapors may not fully react before reaching the desired reaction temperature. Additionally, coke formation can reduce catalyst effectiveness and bio-oil yield. In contrast, ex situ pyrolysis involves the separate pyrolysis of biomass, producing pyrolytic vapors that pass over a catalytic bed in a different reactor (Figure 3). This configuration allows for independent optimization of pyrolysis and catalytic reaction temperatures, thus enhancing catalyst performance. The char produced in the pyrolysis step can be easily separated, improving product purity. However, ex situ pyrolysis requires an additional catalytic reactor, which adds to the costs. Despite this, it generally results in higher selectivity for aromatics and syngas.

Both in situ and ex situ configurations have been shown to significantly improve bio-oil quality. For instance, in situ pyrolysis is more efficient in producing aromatic hydrocarbons, whereas ex situ pyrolysis offers superior performance in terms of bio-oil yield and deoxygenation, particularly at higher temperatures [38,39]. Studies on the in situ catalytic pyrolysis of hemicellulose monosaccharides showed that it promoted furans and acids at lower temperatures, whereas ex situ catalytic pyrolysis reduced these products, favoring dehydration, decarboxylation, and decarbonylation reactions [38]. Moreover, research by Wang et al. comparing in situ and ex situ catalytic pyrolysis of poplar showed that ex situ pyrolysis produced higher yields of olefins, while in situ pyrolysis favored aromatic hydrocarbon formation [40]. Both in situ and ex situ—have their distinct advantages and challenges. The choice of configuration depends on factors such as feedstock type, desired product distribution, and process efficiency. While in situ pyrolysis is simpler and more economical, ex situ pyrolysis offers better control over temperature and product selectivity, especially when aromatics and bio-oil deoxygenation are the key objectives.

### 3.3. Effect of Catalysts

Catalytic pyrolysis has emerged as a promising technique for improving bio-oil yield and quality compared to non-catalytic pyrolysis. While non-catalytic pyrolysis typically produces bio-oil with a high oxygen content, low thermal stability, and high viscosity, catalytic pyrolysis helps to enhance the overall quality of bio-oil by promoting the breakdown of oxygenated compounds into more valuable hydrocarbons, improving fuel properties, and reducing the formation of undesirable by-products such as tar and coke [42]. Catalysts facilitate specific chemical reactions during pyrolysis, leading to more selective product formation and enhancing the yield of bio-oil with lower oxygen content, which is more suitable for further processing or direct use as a fuel [43]. The catalysts commonly used in bio-oil catalytic pyrolysis can be broadly categorized into several types [43]. This paper focuses primarily on zeolite based, carbonaceous materials and metal oxide catalysts, providing a detailed discussion of their impact on bio-oil production and the specific effects on various chemical compounds present in bio-oil.

#### 3.3.1. Zeolite Based Catalyst

**Figure 4:**
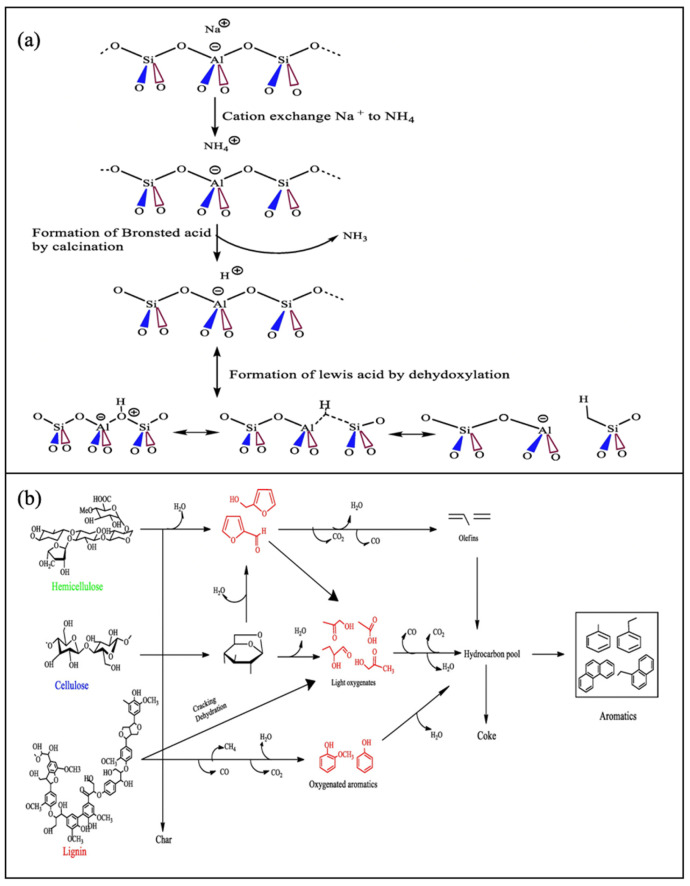
(**a**) Framework of ZSM-5 zeolite catalyst [44]; (**b**) Reaction pathway for catalytic fast pyrolysis over zeolite catalyst [45].

Zeolites are crystalline aluminosilicates characterized by a three-dimensional framework of SiO_4_ and AlO_4_ tetrahedra linked by shared oxygen atoms, forming uniform micropores. This structure imparts high thermal stability, resistance to oxidation, and insolubility in water and inorganic solvents. Zeolites can be categorized based on their origin: natural zeolites, such as clinoptilolite, chabazite, and mordenite, are mined from the earth; synthetic zeolites, like Zeolite A and ZSM-5, are engineered for specific applications, offering uniform pore sizes and tailored properties. Their unique porous architecture enables applications in catalysis, ion exchange, and molecular sieving. Zeolite catalysts, particularly ZSM-5, have been widely recognized for their efficiency in biomass pyrolysis due to their well-defined nanostructural properties, high surface area, and tunable acidity (Figure 4a) [44]. The reaction pathway for catalytic fast pyrolysis over zeolite catalyst is illustrated in Figure 4b [45]. Their three-dimensional microporous framework provides both shape selectivity and strong Brønsted and Lewis acidity, making them highly suitable for upgrading bio-oil and promoting the formation of valuable aromatic hydrocarbons [46,47]. Rahman et al. [48] demonstrated that ZSM-5 selectively promotes aromatization, increasing the yield of aromatic hydrocarbons up to 42.19 wt% during the catalytic pyrolysis of pinewood sawdust. Hu et al. [49] found that HZSM-5 particles facilitated the formation of more olefins in ex situ catalytic pyrolysis. However, one of the main challenges of ZSM-5 is its susceptibility to coke deposition, which significantly reduces its catalytic lifetime. This coke formation can be attributed to the external active sites on the nanoparticle surface, which promote oligomerization and polymerization of small molecules, and to the inherently low H/C ratio of biomass, resulting in hydrogen deficiency during pyrolysis [50]. To address these limitations and improve performance, several nanomodification strategies have been explored. Xue et al. [51] designed a core-shell catalyst (ZSM-5@SBA-15) for maize straw pyrolysis, which significantly enhanced the yield of phenols and hydrocarbons while reducing coke formation, thanks to the improved diffusion and selective catalytic activity offered by the structured shell.

The medium-sized pores (~0.5 nm) within the zeolite structure regulate molecular diffusion and allow precise control over catalytic reactions. While zeolites are effective in bio-oil upgrading, their microporous nature imposes diffusion limitations, particularly when dealing with bulky lignin-derived oxygenates. Studies have shown that micropores restrict the access of larger reactant molecules to the active sites, often leading to increased oxygenate retention, coke formation, and syngas production rather than the desired aromatics [52]. Conversely, zeolites with larger pores, such as beta zeolite (~6 × 7 Å), allow polycyclic aromatic hydrocarbons (PAHs) to form, which are undesired as they act as coke precursors, further reducing catalyst lifespan [53]. Building on this concept, researchers have also examined how pore size influences product selectivity. In catalytic fast pyrolysis of kraft lignin, nano-sized and mesoporous ZSM-5 exhibited enhanced selectivity toward alkylphenols, further reinforcing the impact of pore structure on product distribution [53]. To further optimize diffusion and improve catalyst efficiency, researchers have developed hierarchical zeolites, which integrate micro- and mesoporosity to facilitate reactant diffusion while preserving shape selectivity. Kim et al. [54] reported a significant increase in aromatics production when using hierarchical ZSM-5 and beta zeolite for woody biomass pyrolysis, demonstrating their superior efficiency over conventional microporous zeolites. This finding underscores the importance of tailored pore structures in enhancing product yield, reducing coke formation, and extending catalyst lifetime.

Beyond pore structure engineering, metal doping has been explored as a strategy to enhance the catalytic efficiency of ZSM-5. Zheng et al. [55] investigated the catalytic performance of Zn, Ga, Ni, Co, Mg, and Cu-loaded ZSM-5, and found that metal modifications significantly increased the selectivity for single-ring aromatic hydrocarbons. Among these, Ga-ZSM-5 particles exhibited the strongest deoxygenating ability, followed by Zn- and Ni-modified ZSM-5. Similarly, Mullen et al. [56] reported that K-modified HZSM-5 reduced acidity and increased the yield of alkyl phenols, demonstrating how targeted metal modifications can optimize selectivity and stability.

Table 2 shows the examples of ZSM-5 catalyst in catalytic pyrolysis. It summarized that ZSM-5 and modified ZSM-5 catalysts influence the pyrolysis process by allowing primary vapors to diffuse easily into the pores, where they undergo catalytic cleavage of C–O and C–C bonds, followed by further transformation into small molecules and aromatic hydrocarbons. Modified ZSM-5 catalysts, including metal-loaded and acid/alkaline-treated variants, are more selective towards aromatic hydrocarbons and deoxygenation compared to the parent catalyst, with changes in their structure, acidity, and the number of Brønsted/Lewis acid sites. While repolymerization over the catalysts leads to coke formation and deactivation, metal-loaded ZSM-5 shows improved resistance to coke deposition.

While ZSM-5 remains a benchmark catalyst for bio-oil upgrading, other zeolite-based catalysts have also shown promising results. Studies comparing ZSM-5, mordenite, beta, and Y zeolites revealed that larger-pore beta and Y zeolites allow higher oxygenate diffusion, making them more effective for converting bulky lignin-derived molecules [46]. However, their tendency to form PAHs and coke precursors limits their long-term application. Recent advancements in mesoporous materials like MCM-41, Al-MCM-41, and Fe-MCM-41 have demonstrated the potential for biomass pyrolysis due to their high surface area and tunable acidity. For instance, Casoni et al. [70] observed that Fe-MCM-41 facilitated the production of liquid bio-oil by promoting dehydration reactions while reducing levoglucosan yield, highlighting the importance of acidity control in tailoring product distribution.

#### 3.3.2. Metal Oxide Catalysts

Metal oxides play a crucial role in biomass pyrolysis due to their unique catalytic functionalities, which influence the composition and stability of bio-oil. Their wide availability, cost-effectiveness, and non-toxic nature make them attractive catalysts for bio-oil upgrading. These oxides exhibit distinct reactivity and molecular interactions, enabling them to facilitate deoxygenation, cracking, and selective conversion of bio-derived intermediates [71]. Table 3 shows some examples of metal oxides used in pyrolysis of biomass.

Among the widely studied metal oxides, calcium oxide (CaO) and magnesium oxide (MgO) have demonstrated exceptional performance in modifying bio-oil properties. CaO, known for its strong surface interactions, has been extensively used in reducing bio-oil acidity by promoting the conversion of carboxylic acids into ketones through well-documented condensation and decarboxylation pathways. Additionally, it has been found to capture CO_2_, forming calcium carbonate (CaCO_3_), which helps regulate gas-phase composition by increasing hydrogen and carbon monoxide concentrations [73,75,79,80]. A comparative study by Chong et al. investigated the impact of CaO, MgO, and ZnO in the pyrolysis of fiber-derived fruit biomass. While all three oxides improved bio-oil quality, MgO was particularly effective in reducing levoglucosan content, indicating strong interactions with oxygenated intermediates that promote deoxygenation. However, CaO proved to be the most effective in reducing overall acidity, making it highly suitable for improving bio-oil stability [73]. To further enhance catalytic performance, researchers have explored metal-impregnated CaO catalysts, such as Fe/CaO and Ni/CaO, which introduce additional functionalities that modify molecular pathways. These modifications have been found to improve deoxygenation efficiency and increase aliphatic hydrocarbon yields, thus shifting bio-oil composition toward more energy-dense compounds [81].

Beyond their role in bio-oil stabilization, metal oxides with enhanced molecular interactions have shown great potential in directing specific product selectivity. Transition metal oxides, such as zinc oxide (ZnO), nickel oxide (NiO), zirconium oxide (ZrO_2_), iron oxide (Fe_2_O_3_), and cerium oxide (CeO_2_), have been widely explored for their ability to modulate product distribution by promoting selective reaction pathways [82,83,84,85]. A study by Zhang et al. [77] investigated nine transition metal oxides (CoO, Cr_2_O_3_, CuO, Fe_2_O_3_, Mn_2_O_3_, NiO, TiO_2_, V_2_O_5_, and CeO_2_) in the catalytic pyrolysis of poplar wood. The results showed that cerium, chromium, copper, and iron oxides favored cracking reactions, leading to higher yields of lighter organic compounds, while vanadium, manganese, titanium, and cobalt oxides promoted molecular condensation, increasing the production of heavier organic fractions. Interestingly, NiO exhibited a dual effect, enhancing the formation of both light and heavy organics, indicating its ability to participate in multiple catalytic pathways.

To further enhance catalytic selectivity and efficiency, researchers have developed hybrid metal oxides with tailored properties. Li et al. [86] investigated BaMg-MMO (barium-magnesium mixed oxide) for the selective production of 4-vinyl-phenol (4-VP) from bagasse pyrolysis. It was observed that strong surface interactions in CaO and MgO-based catalysts effectively suppressed undesirable by-product formation, whereas BaO, which exhibited a different catalytic profile, had lower selectivity. The BaMg-MMO catalyst yielded 7.3 wt% of 4-VP, significantly improving the targeted bio-oil fraction compared to non-catalyzed pyrolysis. Further studies on mesoporous metal oxides have shown that their ability to enhance molecular diffusion significantly improves catalytic efficiency. Locatel et al. [87]. examined niobium-based mixed oxides doped with tungsten, aluminum, or manganese in the ex situ conversion of wood pyrolysis vapors. The results revealed that modifications in porosity and surface chemistry allowed for better utilization of catalytic sites. Interestingly, manganese-doped Nb_2_O_5_ catalysts exhibited deoxygenation effects comparable to HZSM-5, further supporting the idea that controlled molecular interactions are key to optimizing bio-oil quality.

Building on this concept, bifunctional catalysts have been developed by combining metal oxides with transition metals to achieve synergistic effects in bio-oil upgrading. Zhang et al. [88] introduced iron into calcium oxide, forming a Ca_2_Fe_2_O_5_-based catalyst for sawdust pyrolysis. This catalyst exhibited an enhanced ability to convert heavy phenols into light phenols, reduce the presence of acidic compounds, and increase the production of furans and aromatic hydrocarbons. The improved performance was attributed to the complementary interactions between CaO and Fe, which facilitated oxygen removal and molecular restructuring. A similar trend was observed by Sun et al. [89], demonstrating the effectiveness of Fe/CaO systems in bio-oil refining. To further expand selectivity and molecular tuning, researchers have investigated the impregnation of noble and transition metals onto oxide supports. Kantarelis et al. [90] evaluated nickel and vanadium supported on silica for vapor-phase deoxygenation and phenol production. While both catalysts enhanced bio-oil quality, Ni-SiO_2_ was particularly selective toward aromatics, whereas V-SiO_2_ effectively reduced acid and ketone content. The in situ reduction of nickel species during pyrolysis was proposed as a key factor in enhancing hydrogen transfer reactions, thus increasing the formation of high-value aromatic hydrocarbons.

#### 3.3.3. Carbonaceous Materials Catalysts

In recent years, carbonaceous materials, including AC, have garnered significant attention in biomass pyrolysis due to their economical and environmentally friendly properties, offering a greener process for cellulose conversion. These materials are often derived from the pyrolysis of biomass or other carbon-rich sources, making them highly cost-effective with minimal chemical inputs required during their preparation [4]. The ability of carbonaceous materials to remain stable in various media, including water, organic solvents, and ionic liquids, allows them to be applied in a variety of pre-treatment processes and catalytic reactions [91]. Typically, these materials are obtained by incomplete carbonization of biomass, followed by sulfonation using concentrated sulfuric acid or chlorosulfonic acid, which introduces phenolic hydroxyl (–OH), carboxylic (–COOH), and sulfonic (SO_3_H) groups. These functional groups play a pivotal role in adsorbing cellulose and breaking the intermolecular hydrogen bonds, making the carbonaceous material highly effective in hydrolyzing biomass components, enhancing its activity for bio-oil production [92].

**Figure 5:**
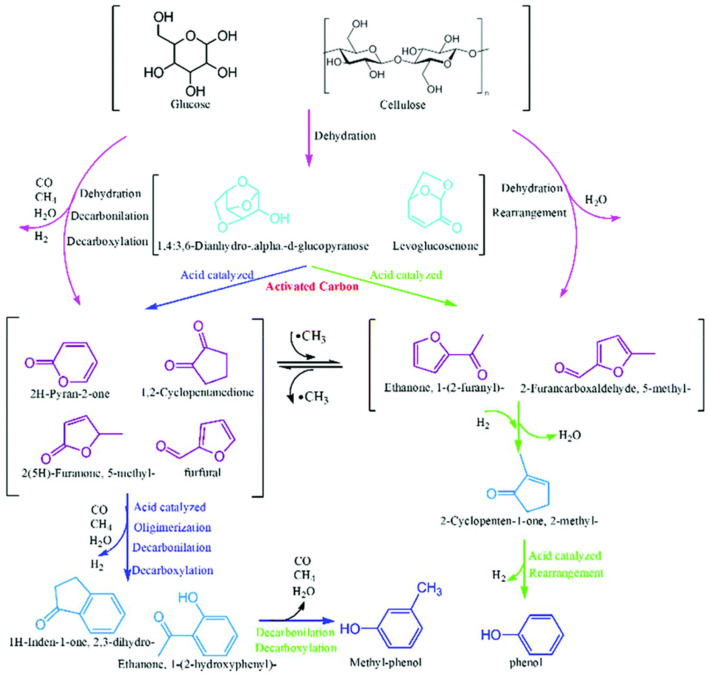
The reaction pathway of phenols production from the catalytic pyrolysis of glucose and cellulose over corn stover-based AC [94].

AC catalysts, derived from biomass or other carbon-rich materials, serves as a product of the pyrolysis process, making them both cost-effective and environmentally friendly, with minimal chemical inputs required during preparation. AC catalysts play a significant role in the selective production of valuable chemicals from biomass pyrolysis. Compared with zeolite-based catalysts, AC offers a more versatile and cost-effective solution for upgrading bio-oil due to its adjustable pore structure and surface properties. The porous network of AC includes micropores, mesopores, and macropores, allowing for the effective conversion of biomass-derived feedstocks into more refined and narrowly distributed compounds. This broader pore distribution helps avoid the steric hindrance commonly encountered in zeolite-based catalysts, which rely only on micropores, thus improving the selectivity and yield of target products, particularly phenols (Figure 5) [93].

Activation is a crucial step in enhancing the surface area, porosity, and reactivity of carbon materials for catalytic applications. AC catalysts, produced through physical or chemical activation, exhibit enhanced catalytic performance due to their well-developed pore structure and surface functional groups. Physical activation typically involves high-temperature treatment (700–1000 °C) under an oxidizing atmosphere (e.g., CO_2_ or steam), creating a well-developed pore structure. In contrast, chemical activation uses agents such as KOH, ZnCl_2_, or H_3_PO_4_ at lower temperatures to introduce functional groups and improve textural properties [95]. By optimizing activation methods, carbon materials can achieve higher catalytic efficiency, making them suitable for bio-oil upgrading and gas-phase reactions in pyrolysis processes.

As a result of these modifications, AC catalysts exhibit remarkable selectivity toward phenols during biomass pyrolysis (Table 4). The production of phenols is primarily attributed to the decomposition of lignin, involving dehydration, decarboxylation, and rearrangement reactions [96]. Studies have shown that commercial AC can achieve a phenols selectivity exceeding 60% in bio-oil, while lignocellulose-based AC exhibits even better catalytic performance and reusability [96,97,98]. In a study by Zhang et al. [94], the catalytic pyrolysis of glucose over a phosphoric acid-activated carbon (ACC) catalyst was investigated to produce phenol-rich bio-oils. The results demonstrated that the selectivity of phenols in the bio-oil could reach up to 100% under optimal conditions, specifically at a reaction temperature of 450 °C with a catalyst-to-reactant ratio of 1. This indicates that ACC is highly effective in converting carbohydrates into phenol-rich bio-oils. Acid activation methods, such as H_3_PO_4_-treated AC, have further enhanced phenols selectivity, while alkali treatments with KOH improve deoxygenation, facilitating the conversion of methoxy-phenols into phenols [99,100].

**Figure 6:**
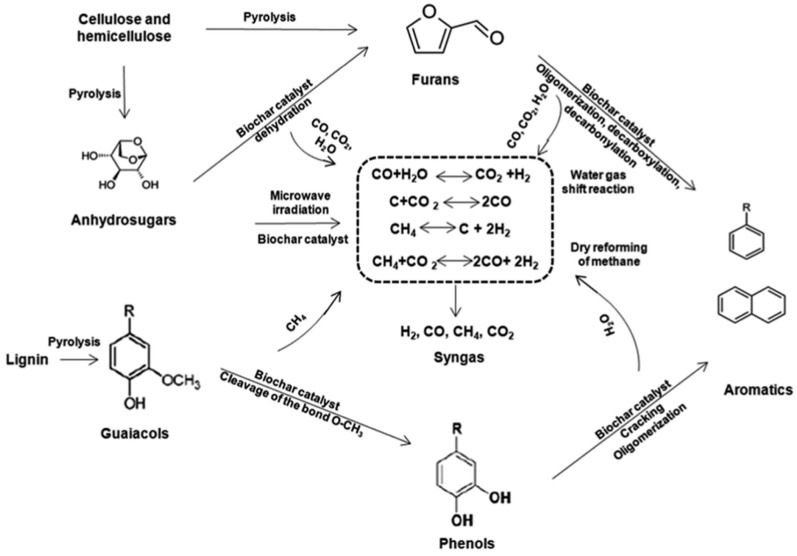
Reaction pathway of biomass catalytic pyrolysis and bio-oil upgrading for hydrocarbon production using biochar catalyst [104].

In addition to phenols, AC catalysts are also highly effective for producing hydrocarbon-rich bio-oil, including alkanes and aromatics (Figure 6). The conversion mechanism involves the transformation of cellulose and hemicellulose into glucose and anhydrosugars, which are subsequently converted into furans and ultimately into aromatics through decarbonylation and oligomerization reactions [104]. Meanwhile, lignin degradation products, such as guaiacols, are converted into phenols and then aromatics through successive cracking reactions. When upgrading bio-oil with hydrogen-rich waste materials, such as plastics, AC catalysts can achieve jet fuel-range hydrocarbons with a selectivity of over 98% [105]. This demonstrates the flexibility of AC in tailoring the composition of bio-oil based on the feedstock and process conditions. More importantly, the catalytic performance of AC can be easily adjusted through simple activation processes, offering a practical advantage over zeolite-based catalysts. Chemical activation with phosphates, such as H_3_PO_4_, not only improves hydrocarbons selectivity but also enhances the recyclability of AC. The incorporation of Fe_3_O_4_ into AC has been explored to create magnetic solid-base catalysts, further facilitating catalyst recovery and reuse [106]. Overall, the combination of low cost, adjustable porosity, and excellent catalytic performance makes AC an attractive alternative for bio-oil upgrading and selective chemical production [93].

**Figure 7:**
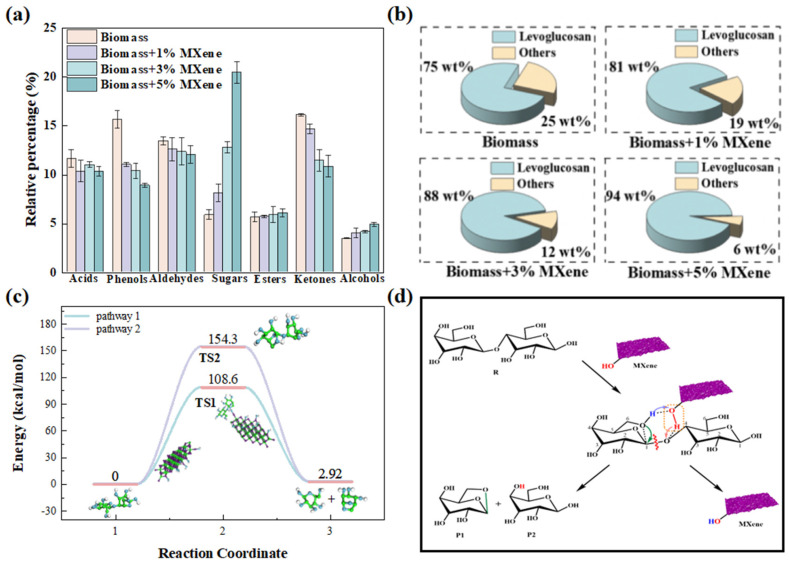
(**a**) Effect of different MXene loadings on bio-oil distribution during pyrolysis; (**b**) Effect of different MXene content on the distribution of LG and other sugars in pyrolysis; (**c**) Formation mechanism of LG and the associated potential energy; (**d**) Schematic diagram illustrating the influence of MXene on levoglucosan formation [108].

After discussing the catalytic properties of AC, another interesting class of catalysts, graphene oxide (GO), has gained attention due to its unique structural and chemical properties. GO is a derivative of graphene that incorporates –OH groups on its surface, which makes it highly reactive and capable of forming strong interactions with biomass components, particularly cellulose. Wang et al. [107] investigated the use of GO as a catalyst for the pyrolysis of poplar wood. The results obtained through Py-GC/MS analysis demonstrated that the addition of GO significantly affect pyrolysis pathways, leading to reduced formation of by-products such as acids, phenols, and aldehydes. More importantly, GO selectively promoted the formation of levoglucosan, a valuable sugar that is a key intermediate in biofuel production. This property of GO allows it to break intermolecular and intramolecular hydrogen bonds in cellulose, promoting the formation of a six-membered ring transition state, which in turn facilitates the production of sugars like levoglucosan during the pyrolysis process. Building on these findings, Wang et al. [108] further explored the use of MXene (Ti_3_C_2_T*_x_*), a 2D material similar to GO, known for its high surface area and unique redox properties, as a catalyst for biomass pyrolysis. Like GO, MXene also features surface functional groups, including –OH groups. The results showed a significant enhancement in sugar yield, particularly levoglucosan, when MXene was used as the catalyst. The MXene catalyst not only boosted the production of levoglucosan but also offered improved performance when compared between in situ and ex situ catalytic pyrolysis, with the latter yielding superior results (Figure 7).

### 3.4. Impact of Nanostructured Catalysts on Bio-Oil Yield

**Figure 8:**
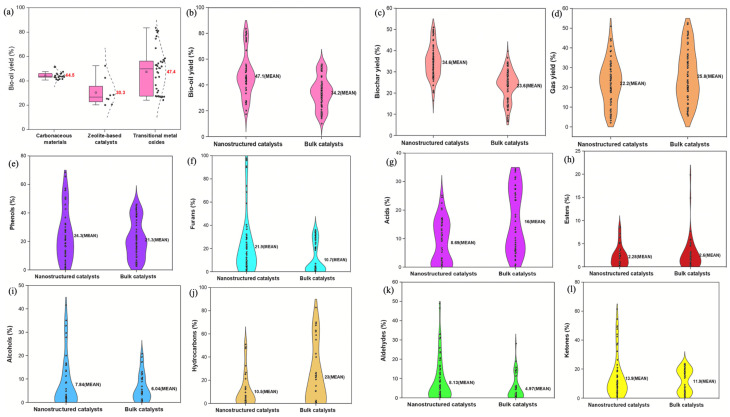
(**a**) Effect of nanocatalyst type on bio-oil yield during biomass pyrolysis; (**b**–**d**) Comparison of product distribution between the nanocatalyst-assisted and bulk catalyst-assisted pyrolysis processes; (**e**–**l**) Comparison between nanocatalysts and bulk catalysts in terms of the product distribution [33].

Building on the discussion of catalyst types and their effects on bio-oil production, some studies have demonstrated the significant impact of nanostructured catalysts on biomass pyrolysis. For example, Lazaridis et al. [53] found that the use of ZSM-5 zeolites in biomass pyrolysis reduced bio-oil yield, with nano-sized ZSM-5 producing the lowest yield (20.3 wt.%) and the highest solids (char and coke). Microporous ZSM-5(40) showed the lowest solids, while mesoporous ZSM-5 offered slight improvements. The results were consistent across both fixed bed and micro-pyrolyzer setups. In addition, as summarized by Shafizadeh et al. [33], the catalytic performance in biomass pyrolysis is greatly influenced not only by the chemical nature of the catalyst but also by its physical structure and scale, whether it is a nanocatalyst or a bulk catalyst (Figure 8). In earlier sections, we discussed the effects of carbonaceous materials, zeolite-based catalysts, and transition metal oxides on bio-oil yield. Here, we expand on how the distinction between nanocatalysts and bulk catalysts can further refine the understanding of product distribution and yield in biomass pyrolysis. The type of nanocatalyst plays a crucial role in determining bio-oil production. Among the tested nanocatalysts, transition metal oxides provided the highest bio-oil yield (47.4%), followed by carbonaceous materials (44.5%) and zeolite-based nanocatalysts (30.3%) [33]. Transition metal oxides generated over 5% more bio-oil than carbonaceous materials and 55% more than zeolite-based catalysts, primarily due to their unique electrical and geometric properties and surface-active crystal facets, which enhance the interaction between reactive species and active sites, resulting in increased bio-oil production [109].

On a broader scale, nanocatalysts exhibit significantly better performance compared to bulk catalysts. On average, nanocatalysts produce 35% more bio-oil (47.1% vs. 34.2%) and 45% more biochar (34.2% vs. 23.6%). In contrast, bulk catalysts yield more syngas, producing over 15% more than nanocatalysts (25.8% vs. 22.2%) [33]. Nanocatalysts exhibit superior catalytic performance in lignin decomposition, increasing the production of phenolic compounds by facilitating the cleavage of α- and β-aryl–alkyl ether linkages and cracking the aliphatic side chains from the aromatic ring [110]. In addition, mesoporous nanocatalysts show higher selectivity toward alkylphenols compared to bulk catalysts. Their deoxygenation capability leads to significantly lower acid content in bio-oil, reducing acid formation by nearly half (8.69% vs. 16%), and a slight reduction in esters (2.28% vs. 2.6%) [111]. However, bulk catalysts produce lower amounts of aldehydes and ketones, with nanocatalysts generating 60% more aldehydes (8.13% vs. 4.97%) and 15% more ketones (13.9% vs. 11.9%), indicating differences in oxygenated compound selectivity between the two types of catalysts [33].

Bulk catalysts and nanocatalysts each offer distinct advantages and limitations in biomass pyrolysis, making them suitable for different applications. Bulk catalysts are more stable and cost-effective, with superior performance in syngas production and oxygenated compound reduction, such as aldehydes and ketones. Their larger particle size and established production methods make them easier to handle and ideal for large-scale operations. However, their lower surface area and limited active sites reduce catalytic efficiency, resulting in lower bio-oil and biochar yields. In contrast, nanocatalysts excel in enhancing bio-oil and biochar yields due to their high surface area and abundant active sites. They are particularly effective in lignin decomposition, promoting the production of high-value compounds like phenols and furans. Nanocatalysts are well-suited for specialized processes that require high selectivity and fine-tuned control, such as advanced bio-oil upgrading. Nevertheless, their preparation is more complex and costly, and they are prone to agglomeration and deactivation under harsh conditions. Each catalyst type plays a crucial role, depending on the desired product and scale of the process.

## 4. Conclusions and Future Work

In conclusion, catalytic pyrolysis presents a promising route for enhancing bio-oil production from biomass. The choice of pyrolysis method—slow, fast, or flash—significantly influences the distribution of products, with fast pyrolysis showing the highest bio-oil yields. The composition of the feedstock, particularly the ratios of cellulose, hemicellulose, and lignin, plays a crucial role in determining the yield and quality of bio-oil. Zeolite, carbonaceous materials, and metal oxides have proven to be the most effective catalysts for improving bio-oil yield and selectivity. Among these, nanocatalysts offer superior performance compared to bulk catalysts, primarily due to their high surface area and enhanced catalytic activity. The combination of nanoengineering with traditional catalysts like zeolites and metal oxides has led to significant improvements in product quality, particularly in the production of phenolic compounds and hydrocarbons. Further research into the structural tuning of catalysts, along with the development of hierarchical zeolites and bifunctional catalysts, is expected to optimize catalytic pyrolysis processes and provide more sustainable, high-yield biofuels.

Future research should focus on advancing catalyst design, particularly through the development of hierarchical zeolites, metal oxide doping, and bifunctional catalysts, to enhance selectivity, stability, and deoxygenation efficiency. Additionally, investigating the fundamental interactions between biomass composition and catalysts will provide deeper insights into reaction mechanisms, leading to improved control over product yields and composition. To ensure the scalability and economic feasibility of catalytic pyrolysis, further studies should evaluate in situ and ex situ reaction conditions, catalyst lifetime, and regeneration strategies. Process integration with feedstock flexibility and recyclability will be essential for adapting pyrolysis to real-world biomass sources. Moreover, techno-economic assessments and life-cycle analysis (LCA) are crucial for evaluating the environmental impact and carbon footprint of catalytic pyrolysis, ensuring that it aligns with sustainable biofuel production goals. Ultimately, optimizing catalytic pyrolysis will pave the way for its commercial application in biofuel and bio-based chemical production, offering a viable alternative to fossil fuels and contributing to the development of a circular bioeconomy.

## Figures and Tables

**Table 2:** ZSM-5 catalysts in catalytic pyrolysis.

Feedstock	Catalyst	Pore Size (nm)	BET (m^2^/g)	Selectively	Refs.
Pinewood sawdust	ZSM-5	/	350	Aromatic hydrocarbon	[48]
Sawdust	Metal modified ZSM-5	3.88–4.19	248.46–288.33	BTX production	[57]
Rice straw	Hexadecyl trimethyl ammonium bromide modified hierarchical HZSM-5	2–8	333–462	Aromatics	[58]
Rape straw	Parent and hierarchical HZSM-5	3.3–7.6	/	Aromatic hydrocarbons	[59]
Soybean straw and soapstock	ZSM-5 and composite catalyst	3.77 and 42.915	290.76 and 6.004	Aromatics	[60]
Corn stover and low-density polyethylene	HZSM-5	/	425	Hydrocarbon	[61]
Torrefied rice husk	Parent and Fe-modified ZSM-5	2.014–2.024	334.81–381.82	Upgrading bio-oil	[62]
Sewage sludge	Ni and Co loaded HZSM-5	4.88–5.12	258–306	Aromatic rich bio-oil	[63]
Green microalgae	Mg-Ce/ZSM-5	1.418–1.924	110.08–311.69	High quality bio-oil	[64]
Cellulose	Parent and metal-modified hierarchical HZSM-5	/	156–366	Aromatics	[50]
Cellulose	ZSM-5 and dual catalyst bed of CaO and ZSM-5	3.49	363.7	Light olefins and aromatics	[65]
Lignin	HZSM-5	/	425	Upgrading of pyrolytic vapor	[66]
Hydrolysis lignin	ZSM-5 and other zeolite catalysts	1.3	425	Hydrocarbon rich bio-oil	[67]
Hydrolysis lignin	Acid-modified and Ni-loaded ZSM-5	1.3–1.6	381–401	Mono-aromatics/phenol	[68]
Ethanol and oleic acid	Mesoporous ZSM-5	4.8–22	405–469	Olefin production	[69]

**Table 3:** Metal oxide catalysts for biomass pyrolysis.

Biomass	Catalysts	BET (m^2^/g)	Selectively	Refs.
Cotton stalk	CaO	3.20	Furans and carboxylic acids (decreased)	[72]
Palm empty fruit bunches	MgO	19.84	Levoglucosan (decreased)	[73]
Forest residues	CaO and MgO	1.97 and 8.14	Deoxygenation	[74]
Oakwood	CaO	8.7 ± 0.2	Ketones and light phenols	[75]
Switchgrass	CaO	/	Phenols and hydrocarbons	[76]
Poplar wood	CoO, Cr_2_O_3_, CuO, Mn_2_O_3_, NiO, TiO_2_, V_2_O_5_ and CeO_2_	/	Alcohol, furans, ketones, acetic acid and phenolic compounds	[77]
Wood	CaO and CaO/MgO	/	Decreased acidity and oxygen content	[78]

**Table 4:** The phenols selectivity from pyrolysis over AC catalysts.

Biomass	Catalyst	BET (m^2^/g)	Bio-Oil (%)	Selectivity (%)	Refs.
Palm kernel shell	Commercial AC	707.65	45	64.58	[101]
Glucose	AC with H_3_PO_4_ activation	1125.6	49.6	100	[94]
Douglas fir sawdust	AC with H_3_PO_4_ activation	559.82–889.67	25	43.24	[102]
Lignin	AC with H_3_PO_4_ activation	/	27	95.5	[103]
Acid pretreated lignin	AC with H_3_PO_4_ activation	/	22.3	98.2	[103]
Douglas fir pellet	Lignite coal-DARCO 830	/	28.97	74.77	[97]
Wood-DARCO MRX	Douglas fir pellet	/	26.5	74.61	[97]
